# The Metabolic Interplay between Plants and Phytopathogens

**DOI:** 10.3390/metabo3010001

**Published:** 2013-01-08

**Authors:** Guangyou Duan, Nils Christian, Jens Schwachtje, Dirk Walther, Oliver Ebenhöh

**Affiliations:** 1 Max Planck Institute for Molecular Plant Physiology, 14476 Potsdam, Germany; E-Mails: duang@mpimp-golm.mpg.de (G.D.); schwachtje@mpimp-golm.mpg.de (J.S.); 2 Institute for Complex Systems and Mathematical Biology, University of Aberdeen, Aberdeen City AB24 3UE, Aberdeen, United Kingdom; E-Mails: n.christian@abdn.ac.uk (N.C.)

**Keywords:** plants, pathogens, metabolic networks, genome, enzymes, metabolites, metabolic impairment, visualization

## Abstract

Plant diseases caused by pathogenic bacteria or fungi cause major economic damage every year and destroy crop yields that could feed millions of people. Only by a thorough understanding of the interaction between plants and phytopathogens can we hope to develop strategies to avoid or treat the outbreak of large-scale crop pests. Here, we studied the interaction of plant-pathogen pairs at the metabolic level. We selected five plant-pathogen pairs, for which both genomes were fully sequenced, and constructed the corresponding genome-scale metabolic networks. We present theoretical investigations of the metabolic interactions and quantify the positive and negative effects a network has on the other when combined into a single plant-pathogen pair network. Merged networks were examined for both the native plant-pathogen pairs as well as all other combinations. Our calculations indicate that the presence of the parasite metabolic networks reduce the ability of the plants to synthesize key biomass precursors. While the producibility of some precursors is reduced in all investigated pairs, others are only impaired in specific plant-pathogen pairs. Interestingly, we found that the specific effects on the host’s metabolism are largely dictated by the pathogen and not by the host plant. We provide graphical network maps for the native plant-pathogen pairs to allow for an interactive interrogation. By exemplifying a systematic reconstruction of metabolic network pairs for five pathogen-host pairs and by outlining various theoretical approaches to study the interaction of plants and phytopathogens on a biochemical level, we demonstrate the potential of investigating pathogen-host interactions from the perspective of interacting metabolic networks that will contribute to furthering our understanding of mechanisms underlying a successful invasion and subsequent establishment of a parasite into a plant host.

## 1. Introduction

Photosynthetic organisms form the basis of all food webs and higher land plants are the primary energy and carbon source for terrestrial ecosystems and fundamental to feed the human population. However, all of the approximately 300,000 plant species regularly suffer pathogen and herbivore attacks [1]. The annual yield of crop plants is severely diminished by the regular outbreaks of plant diseases, a large part of which is caused by pathogenic fungi and bacteria. Considering that some of these pathogens are estimated to account for a loss in crop yield that could feed tens of millions of people, the socio-economic impact of microbial plant pathogens cannot be overestimated. Thus, the need for a systematic and comprehensive understanding of the detrimental impacts of pathogens on plants is evident.

Several plant-pathogen systems are increasingly well understood at the molecular level, including the complex signaling pathways that orchestrate the various defense responses of plants. The interactions of host plants with their bacterial and fungal pathogens are described by using a “zigzag” model that consists of pathogen-associated molecular pattern-triggered immunity (PTI) and, subsequently, effector-triggered immunity (ETI) that depends on effector proteins that are secreted by the pathogen and recognized by plant cells [2]. Even though there is much progress made in examining the underlying molecular events, insights in the regulation and changes of plant metabolism during pathogen invasion are only recently emerging, supported by new sophisticated methods for metabolite analyses. Thus far, the description of metabolism has been targeting almost exclusively the plant side of the interaction (e.g. [3]) with few studies using mix-cell cultures to distinguish between plant and pathogen metabolism (e.g. [4]). 

After infection, when the pathogen has established itself in the host, it will heavily depend on host metabolism and, as a consequence, the metabolism of pathogen and host become tightly interlinked. This generally imposes severe nutrient losses of the plant to the pathogen. It has been shown that several bacterial and fungal pathogens are able to manipulate host metabolism, e.g. sucrolytic enzymes, such as cell wall invertase, to turn the infected tissue into a carbohydrate sink that provides hexoses to the pathogen [5]. However, pathogens exhibit different life-styles and colonize various tissues of plants, where they differentially interact with plant cells. Some biotrophic bacteria colonize the apoplast and feed on nutrients of the apoplastic fluids, whereas some biotrophic fungi establish structures inside of the host cells (haustoria) that allow an in-cell nutrient exchange. After a biotrophic phase, hemi-biotrophic pathogens eventually destroy plant cells to feed on the remnants, as necrotrophic pathogens do [6]. Furthermore, it has been shown that several obligate pathogens and symbionts have lost parts of their metabolic networks, because products of the respective proteins are continuously supplied by the plant host [7,8] and latest studies indicate that this process is rather driven by genetic drift than by selection [9]. Thus, metabolic networks of obligate pathogens may lack crucial reactions.

With regard to molecular interactions in host-pathogen systems, previous studies have investigated the gene expression changes in several host-pathogen systems [10,11] especially to investigate the host’s defense mechanism. Integrated *in silico* metabolic models have also been created with the goal to characterize the pathogen’s metabolism in the host-environment, e.g. bacteria in mammalian hosts [12,13]. However, large-scale metabolic analyses considering multiple hosts (especially plants) and pathogens together have not been reported yet as whole-genome scale metabolic models had not been available yet. Previous studies have tried to overcome this limitation by incorporating transcriptomics data [14]

Rapid advancement in genome sequencing [15] and annotation [16,17] made possible the study of interactions between plants and related pathogens from the genome-wide perspective. We used the published genome sequences of five plant species and those of associated five parasitic microbial organisms to derive models of their metabolic networks. As a central selection criterion for the plant-pathogen pairs investigated here, we required that the complete genomic sequence is available to allow for a genome-wide annotation of all proteins, and thus, enzymes and associated metabolic reactions. The derived network models are intended to serve as a basis for future theoretical investigations of the metabolic interactions between plants and phytopathogens. As a first approximation, we assumed free exchange of nutrients between host and pathogen. This is, of course, a simplification, since membranes are present between pathogen and host, and pathogens are known to specifically employ amino acid and sugar transporters to gain access to nutrients [18], or may modify host cell membrane structure to alter nutrient leaking into the apoplast. 

We present an intuitive graphical interface, which allows for an easy, graphics-supported inspection of interacting plant-pathogen network pairs. This tool is intended to give microbiologists and biochemists the possibility to visualize and manually explore the network interactions, as we expect increasing availability of genomic and metabolic data of organismic interactions. We then present some initial analyses of the effects of merging two metabolic networks. In particular, we analyze and quantify positive and negative effects of one network on another when merged. Both the positive and negative effect measures are based on the notion of the 'metabolic scope' [19], which is defined as the set of metabolites an organism is in principle capable of producing if a defined combination of nutrient metabolites is present. For the positive measure, we calculate the metabolic gain [20] describing by how many metabolites the biosynthetic potential of a pair of networks is increased compared to the sum of the two networks in isolation. As a negative measure, we introduce the 'metabolic impairment', which quantifies the impact of a pathogen on the ability of the host plant to produce necessary biomass precursors. This approach was also inspired by studies on interacting bacterial communities that investigated their effective metabolic overlap [21]. By focusing on the mutual gain as well as impairment resulting from pathogens invading the plant host, our study complements other approaches that investigated the mutual biosynthetic support in parasite-host relationships from a metabolic network perspective [22,23]. Our emphasis lies specifically on the consequences of the interaction rather than the mutual metabolic “input” requirements. With our study, we wish to further illustrate the potential of approaching plant-pathogen interactions from a metabolic network perspective.

## 2. Methods

### 2.1. Plant-Pathogen pairs, Sequence Information

In this project, five pairs of plant-pathogen were selected (Table 1). The rust fungus *Melampsora larici-populina* is a major threat in European poplar plantations [24]. Biotrophic rust fungi are some of the most devastating pathogens of crop plants. The biotrophic bacterium *Xanthomonas oryzae* pv*. Oryzae* causes bacterial blight of rice (*Oryza sativa L.*), which can cause reductions of rice yields of as much as 50% in some areas [25]. The necrotrophic fungal pathogen *Sclerotinia sclerotiorum* causes stem rot or white mold on soil-borne plants in more than 500 species of plants globally [26], including the important feedstock soybean (*Glycine max)*. The plant model species *Arabidopsis thaliana* is infected by the hemi-biotrophic pathogen *Pseudomonas syringae* pv*. Tomato* [1] and this interaction is widely used to study the underlying mechanisms of pathogen attack [6]. The biotrophic fungi *Ustilago maydis* can cause smut disease in maize [27], even though this pathogen is not a major pest in crop plants. All protein sequences used to build the species-specific metabolic networks for all 10 species were downloaded from the NCBI protein database (see description in Table 2). The non-redundant “NR” database used in the BLAST [28] analysis was downloaded as of Jan 9, 2011.

**Table 1:** Overview of the selected plant-pathogen pairs investigated in this study and associated key biological aspects. NCBI taxonomy numbers are given in the parentheses next to the species’ NCBI Taxonomy names.

Pathogen	Plant	Pathogen type	Unicellular/multicellular	Tissue colonisation	Obligate pathogen
*Pseudomonas syringae* pv. *tomato* (323)	*Arabidopsis thaliana* (3702) [1]	Bacterium/hemi-biotrophic [6]	unicellular	apoplast	no
*Xanthomonas oryzae* pv. *Oryzae*(64187)	*Oryza sativa* (4530) [25]	Bacterium/biotrophic [29]	unicellular	apoplast	no
*Ustilago maydis* (5270)	*Zea mays* (4577) [27]	Fungus/biotrophic [27]	multicellular	apoplast and cells	yes
*Melampsora larici-populina* (203908)	*Populus trichocarpa* (3694) [30]	Fungus/biotrophic [30]	multicellular	apoplast and cells	yes
*Sclerotinia sclerotiorum* (5180)	*Glycine max* (3847) [31]	Fungus/necrotrophic [32]	multicellular	apoplast	no

**Table 2:** The number of protein sequences in the investigated organisms (downloaded from NCBI as of July 2011). The second column lists the abbreviations for each organism used in the following parts of the article.

Organism pair	Abbreviation	Number of proteins
*Arabidopsis thaliana* [33]	At	221,677
*Pseudomonas syringae* pv*. Tomato* [34]	Ps	41,274
*Oryza sativa* [35]	Os	257,407
*Xanthomonas oryzae* pv.* Oryzae* [25,36]	Xo	29,011
*Zea mays* [37]	Zm	101,421
*Ustilago maydis* [38]	Um	14,433
*Populus trichocarpa* [39]	Pt	87,553
*Melampsora larici-populina* [40]	Ml	16,384
*Glycine max* [41]	Gm	35,645
*Sclerotinia* * sclerotiorum*	Ss	30,901

### 2.2. Species-Independent Reaction Models

**Figure 1:**
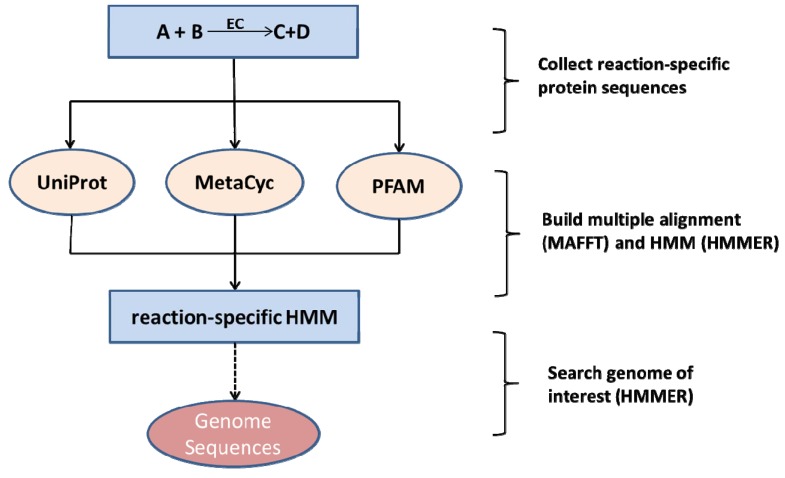
Enzyme annotation workflow applied in this study. For every reaction, enzyme sequences were extracted from databases and used to build reaction-specific and species-independent profile hidden Markov models (HMMs). Using the HMMER software, these reaction HMMs are then used to scan the organism’s protein sequence set resulting in E-values that reflect the probability that a particular protein acts as an enzyme and catalyzes the reaction that is captured by the specific HMM [47].

We built draft genome-wide metabolic networks for each plant and its pathogen based on the MetaCyc reference metabolic network [42], and the protein databases Pfam [43], UniProt [44], and NCBI. The annotation of enzymes in the genomes of the 10 species investigated here was based primarily on applying hidden Markov models (HMMs). First, all reactions from MetaCyc with at least one annotated protein sequence were extracted. If for such a reaction a corresponding Pfam domain was annotated, the associated profile-HMM as provided by Pfam was used for further computations. If no Pfam family was annotated, but a four-digit EC number for the reaction was given, all protein sequences annotated with the corresponding EC number were downloaded from UniProt and used to build HMM-models (see below). If fewer than 20 protein sequences were available for a given EC number, standard BLAST [28] with a score cut-off of 50 and an *E*-value threshold of 10^-10^ was used to collect additional significant sequence hits from the non-redundant sequence database “NR” to allow creating HMM-models with a sufficient number of sequences. 591 HMMs were derived from Pfam, 1192 HMMs from UniProt associations with EC-numbers, and 399 HMMs needed additional protein sequences obtained by BLAST searches. Afterwards, all reaction-specific protein sequences were aligned using the multiple sequence alignment program MAFFT [45]. The resulting multiple sequence alignments were converted into a reaction-specific profile HMM using the HMMER program [46]. HMMER transforms a multiple-sequence alignment into a probability based position-specific scoring system. Finally, all protein sequences from each species were searched with HMMER using all reaction-specific HMMs. For every reaction HMM, the protein with the lowest *E*-value score and below the cut-off of 1 was assigned the annotation associated with the respective HMM. The workflow is shown in Figure 1. Thus, a given HMM is assigned to only one protein in a species’ genome at most. While in reality a particular enzyme may exist in multiple isoforms, for the purpose of network reconstruction a reaction can proceed as long as there is at least one enzyme catalyzing it. However, a single protein may carry more than one annotation as it may be identified as the best hit by more than one HMM. First, proteins may indeed carry out multiple reactions (e.g. acting on different substrates), and secondly, using the bioinformatics annotation protocol alone, ambiguous assignments cannot be easily resolved, but would require experimental verification. For the sake of network capacity, we decided to accept ambiguous, but significant assignments.

### 2.3. Network Curation and Gap-Filling

The draft metabolic network models derived by the method explained in the previous section were further curated by removing reactions that are stoichiometrically inconsistent in the sense that the sum formulas on both sides of the chemical equations do not yield the same number of atoms. Mass-balance was checked in reactions for which all involved compounds are annotated with a chemical formula. If elements were net-produced or net-consumed violating mass conservation (neglecting protonation state and water production/consumption), the reaction was removed. Reactions marked spontaneous in the MetaCyc database were added to all organism-specific metabolic networks. The resulting plant networks were then extended using the method introduced in [47]. Briefly, the metabolic networks are assumed to produce essential biomass precursors (amino acids, nucleotides) from autotrophic nutrients consisting of carbon dioxide, water, oxygen, protons and all other inorganic metabolites found in the MetaCyc database. Further, we added ribulose bisphosphate to ensure the functioning of the Calvin cycle. If the draft network is not able to produce the essential biomass precursors, a greedy approach identifies reactions from the MetaCyc database that should be added to the network to fulfill this requirement. It was not possible to apply this approach to the pathogen networks, because their nutrient requirements are presently largely unknown.

### 2.4. Interaction Analysis of Metabolic Networks

When two metabolic networks interact, they can exhibit positive or negative effects on each other. If the networks “cooperate” and combine their biochemical resources, increased biosynthetic capabilities may result from the interaction. A concept to quantify this symbiotic effect was introduced in [20], where the ‘metabolic gain’ was defined. The gain exists for any combination of initially available metabolites, called the seed. To calculate the gain, two networks are combined by simply forming the union of their biochemical reactions. Then, the scope of the seed, which is defined as the set of metabolites that can be produced from the seed as determined by the method of network expansion [19], is calculated for the individual networks and the combined network. The gain is then defined as the number of metabolites in the scope of the combined network minus the number of metabolites in the union of the scopes of the single networks. The asymmetric gain describes the positive effect on the individual organism, by subtracting the number of metabolites in the scope of a single network from the number of metabolites in the scope of the combined networks intersected with all compounds from the single network. To calculate the gain, we used as seed the combination of nutrients on which the plant networks can grow (see above), *i.e.* produce all necessary biomass precursors.

Calculating negative effects of one network on another is less straightforward than capturing positive effects. To measure how the pathogen can impair the plant network, we first determined minimal combinations of nutrients on which the pathogen can survive (i.e. produce all necessary biomass precursors). Typically, the minimal nutrient seed compound set consisted of three to six compounds depending on the pathogen. In addition, water, oxygen, and protons were always provided. We employed the method described in [48] with the modification that the nutrient compounds are restricted to metabolites present in the plant network. In this way, possible nutrient combinations, which the pathogen may extract from the plants, were determined. The method was based on a statistical sampling procedure and is therefore repeated 10,000 times for each pathogen network. To favor small molecules as nutrients, the sampling was not performed according to a uniform random number distribution, but by using a Boltzmann-factor-based shuffling procedure (for details see [49]), using the compound masses, *m*, as "energies" and a “thermodynamic beta” of 0.05 1/Da. Two compounds with the mass difference Δ*m* are then exchanged with the probability of *min*(e^-beta*^^Δ*m*^,1)*.* For each solution, *i.e.* a set of nutrients on which the pathogen can thrive, the possible negative effect on the plant network caused by the withdrawal of the corresponding metabolites was determined as follows: All reactions that use as substrate at least one of the determined pathogen nutrient compounds were removed from the plant network. This corresponds to the extreme scenario in which the pathogen extracts the entire compounds from the plant and reactions requiring this compound in the plant are no longer possible. Employing network expansion [19], this reduced network was then used to determine, which biomass compounds that are essential for the plant can still be produced. The relative impairment is then defined as the fraction of minimal nutrient combinations, for which a particular essential biomass compound can no longer be produced. For example, a relative impairment of 0.3 for the plant biomass component lysine means that in 30% of all calculated minimal nutrient combinations one or several nutrients have been removed by the pathogen from the plant, which render lysine not producible. It is in the nature of this analysis that only the effect of the pathogen on the host is evaluated. Therefore, no ‘symmetric’ score, as in the case of the positive effects discussed above, exists.

Vectors containing the impairment scores were defined to characterize impairment patterns of host-pathogen interactions and pairwise distances between these vectors were calculated. Based on these distances, we applied Multidimensional Scaling (MDS) to visualize similarities of the impairments in a two-dimensional diagram. The 'cmdscale' routine of R was used for the MDS computations.

### 2.5. Network Comparison and Multidimensional Scaling (MDS)

The metric for the comparison of metabolic networks was based on Jaccard coefficients (JC) applied to sets of present reactions. The Jaccard coefficient between two sets is defined as the number of elements in the intersection divided by the number of elements in the union of the two sets. It is one for identical sets and zero for completely disjoint sets. We defined the Jaccard distance between two networks as *d*(*N*1,*N*2) = 1-*JC*(*N*1,*N*2), where *JC* is the Jaccard coefficient as defined above.

### 2.6. Network Visualization

For all five pairs of plant and pathogen, we mapped the reactions onto the KEGG [48] reference metabolic pathway map based on the MetaCyc annotation. If there was no corresponding KEGG reaction annotation in MetaCyc, the EC number was used to map between KEGG and MetaCyc. Using the KEGG html-based visualization, all pathway maps can be zoomed in and out and can be queried. All metabolic network maps for all five plant-pathogen pairs are available as Supplementary Material 1; the related reactions for all the species are available as Supplementary Material 2.

## 3. Results

### 3.1. The Genome-Scale Metabolic Networks

Our established annotation workflow (see Figure 1) resulted in 10 genome-scale metabolic draft networks, five representing the metabolism of plants and five the metabolism of phytopathogens, respectively.

The applied extension method suggested adding one reaction (catalyzed by imidazoleglycerol phosphate synthase) to all plant networks in order to produce histidine. No alternative reactions were found. Because no sequence information for enzymes catalyzing this reaction is available, a closer inspection whether these enzymes are indeed coded in the genome was not possible. For *Arabidopsis thaliana*, several reactions were additionally suggested in order to produce thymidine triphosphate (TTP). For the metabolic networks of *Glycine max* and *Zea Mays*, more reactions had to be added to enable the network to produce required biomass precursors from carbon dioxide and inorganic material. The numbers of added reactions are given in Table 3.

**Table 3:** Statistics of the number of reactions (annotated and after removing stoichiometrically inconsistent ones (‘curated’) and number of metabolites (connected to curated reactions) in all 10 organisms studied. The percentage values in the fourth column represent the percentage of curated reactions. In the final column, the numbers of reactions added to the plant networks during the gap filling process are denoted. This is not applicable (NA) to the pathogenic networks.

Organism	Kingdom	No. reactions (annotated)	No. reactions (curated)	No. metabolites (curated)	No. of added extension reactions
At	Planta	3,608	3,316 (91.9%)	3,560	2
Ps	Bactera	3,223	2,964 (92.0%)	3,175	NA
Os	Planta	3,680	3,357 (91.2%)	3,617	1
Xo	Bacteria	3,026	2,799 (92.5%)	3,064	NA
Zm	Planta	3,606	3,315 (91.9%)	3,596	4
Um	Fungi	3,398	3,107 (91.4%)	3,398	NA
Pt	Planta	3,758	3,442 (91.6%)	3,653	1
Ml	Fungi	3,368	3,084 (91.6%)	3,356	NA
Gm	Planta	3,380	3,130 (92.6%)	3,446	4
Ss	Fungi	3,505	3,200 (91.3%)	3,493	NA
MC		9,531	8,780 (92.1%)	7,755	NA

The sizes of the networks, measured in terms of numbers of reactions and numbers of metabolites, are summarized in Table 3. On average, plant networks are larger than pathogen networks (*p*_*t-test*_ = 0.01) with 3082 curated reactions present on average in plants, 2906 in fungi, and 2690 in bacteria. Despite its significance, the difference in size of the plant and bacterial metabolic networks is surprisingly small. It has to be borne in mind that the employed network reconstruction procedure relied on mapping known enzyme sequences onto novel protein sequences. As a consequence, these networks are biased to well-known and common enzymatic reactions. Thus, due to limitations in our current knowledge, the large secondary metabolism of plants is hugely underrepresented.

**Figure 2:**
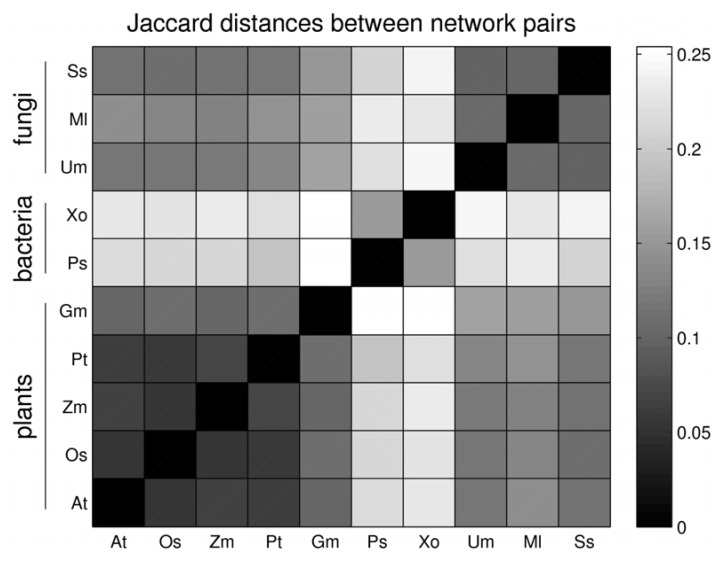
Pairwise network-network overlap based on the Jaccard distance for present metabolic reactions in the 5 plant and 5 phytopathogen genomes investigated in this study. The values of the respective Jaccard indexes are visualized by grey-scale.

Beyond the size differences, the question whether the content of the networks are rather similar or display significant differences; *i.e.* whether the set of enzymes and reactions are overlapping or disjoint. Judged by the Jaccard distance of present reactions as a measure to compare two metabolic networks (see Methods), the five plant metabolic networks are most similar to each other (Figure 2), whereas the pathogen networks are much more heterogeneous. The metabolic networks of the bacterial pathogens (*Xo* and *Ps*) clearly differ from those of fungal species (*Um*, *Ml*, *Ss*) with the bacterial species representing the most distinct networks compared to all other species considered here. In fact, the metabolic reaction-based similarities of the networks reflect the respective evolutionary origin of the 10 different species (Figure 2). 

### 3.2. Visualization of Pathogen-Plant Metabolic Networks

A central goal of our work was to establish draft networks for various plant-pathogen pairs, which may offer a platform for further investigations. To facilitate the inspection of how the host and the pathogen networks are overlapping and interacting, we generated navigable pathway maps that are based on the KEGG [48] metabolic maps. As an example, Figure 4 displays the visualization of the interacting network pair *Pseudomonas syringae* pv. *Tomato and Arabidopsis thaliana.* Green edges represent *Arabidopsis thaliana* specific reactions, yellow edges represent *Pseudomonas syringae* pv. *Tomato* specific reactions, black reactions are common to both networks. Grey edges represent reactions not contained in any of the two species. All network pairs are accessible as interactive maps with links to the corresponding KEGG metabolite and reaction entries in the Supplementary Material 1.

**Figure 3:**
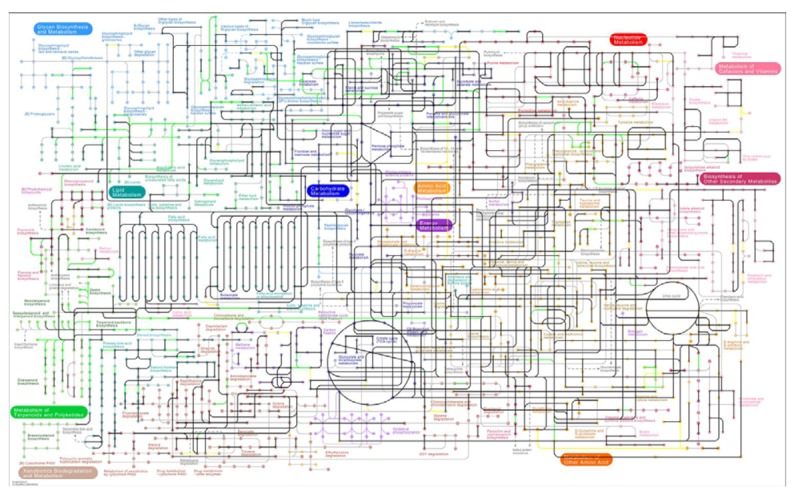
Interacting metabolic networks of *Pseudomonas syringae* pv. *Tomato* and *Arabidopsis thaliana* based on KEGG. Black edges represent common reactions between *Pseudomonas syringae* pv. *Tomato* and *Arabidopsis thaliana*, green edges represent *Arabidopsis thaliana* specific reactions, yellow edges represent *Pseudomonas syringae* pv. *Tomato* specific reactions, all other edges represent reactions not contained in either organism.

### 3.3. Network Analysis of Plant-Pathogen Network Pairs

The pathogen gaining its resources from the host, thus inflicting damage to it, constitutes the hallmark of any host-pathogen relation. This relation is asymmetric in the sense that the host can survive better in the absence of the pathogen, while in general, the pathogen is dependent on nutrients or other components provided by the host. At the metabolic level, it is therefore plausible to assume that the presence of the metabolism of the pathogen has a negative effect on the plant metabolism, whereas the existence of the plant metabolism is positive for the pathogen. To analyze and quantify negative and positive effects of interacting metabolic networks, we calculated a ‘gain’ measuring the positive effect and an ‘impairment’ measuring the negative effect that one network has on the other. Essentially, the gain measures the number of metabolites, which can be produced more by a combined network compared to the sum of the single networks and the impairment is assessed by calculating the negative effect that removal of required nutrients of the pathogen has on the host’s capability to produce essential biomass precursors (details are given in the relevant section in Methods). 

We calculated the metabolic gain for each plant-pathogen pair by assuming the photoautotrophic plant seed consisting of carbon dioxide and inorganic nutrients. As described in the relevant paragraph in the Methods, we calculate a symmetric gain, which describes the overall increase of producible metabolites, and asymmetric gains, which specify the advantages for each partner by only including metabolites occurring in the network of the respective interacting partner. This plant asymmetric gain describes how many new compounds could in principle be synthesized by the plant if all metabolic reactions in the pathogen could be used constructively. While this may seem irrelevant considering that pathogens do not help their hosts but rather exploit them, it is nevertheless an interesting theoretical exercise, because a transition from pathogenic exploitation to symbiotic mutualism can occur, and vice versa. For example, type III secretion systems that are crucial for the pathogenicity of *P. syringae*, are also active in the plant growth promoting *Pseudomonas fluorescens* SBW 2528 [50], and haustoria are established by pathogenic fungi, e. g. powdery mildew, as well as by symbiotic mycorrhiza (here called arbuscules). Some symbionts may have evolved from pathogens and adapted to their hosts by providing them with valuable chemical substances [51]. It is plausible to assume that pathogens, at least to some degree, may also be physiologically beneficial to their host’s metabolism. 

The plant asymmetric gain varies considerably for the five pairs. By far the highest value is observed for *Glycine max* with *Sclerotinia sclerotiorum*. Therefore, the metabolism of the parasite *S. sclerotiorum* appears to have a high potential to be of use for its host. It can be speculated that in evolutionary terms this parasitism has some chance to evolve into a symbiotic relationship, because both partners can – at least at the metabolic level – in principle benefit considerably from each other. Except for one pair (*Gm/Ss*) where it is relatively even, the asymmetric gain is considerably higher for all pathogens relative to the plant hosts in all pairs. This is not surprising considering that we performed our calculation under the assumption that only inorganic nutrients are available. Clearly, the parasites cannot utilize this combination without presence of the plants. Interestingly, the symmetric metabolic gain and the plant asymmetric gain do not seem to be correlated with network distance. Intuitively, one would expect that the larger the network overlap (small Jaccard distance), the smaller the gain, because the networks have essentially the same metabolic capacity, and conversely, the smaller the overlap (large Jaccard distance), the larger the gain. However, no significant correlation, even slightly negative rather than the expected positive correlation, was found between the gain or asymmetric gain and the Jaccard distance (with the Pearson correlation coefficients and associated p-values of *r=*-0.25, *p*=0.68, and *r*=-0.10, *p*=0.87, respectively). For example, *Glycine max* and *Sclerotinia sclerotiorum* exhibit the largest gain amongst the five pairs (191 (symmetric), 238 (asymmetric)), whereas the network distance of 0.145 is rather moderate. By contrast, *Arabidopsis thaliana* and *Pseudomonas syringae* exhibit a rather large Jaccard distance of 0.216, but the metabolic gain is only 1 (symmetric) and 34 (asymmetric).

**Table 4:** Overview of the metabolic gain and Jaccard distance for all five plant-pathogen pairs.

	gain	asymmetric gain plant	asymmetric gain pathogen	Jaccard distance
*At - Ps*	1	34	146	0.216
*Gm* *– Ss*	191	238	220	0.145
*Os* *– Xo*	21	68	214	0.223
*Pt* *– Ml*	2	14	301	0.140
*Zm* * –Um*	8	19	308	0.122

**Figure 4:**
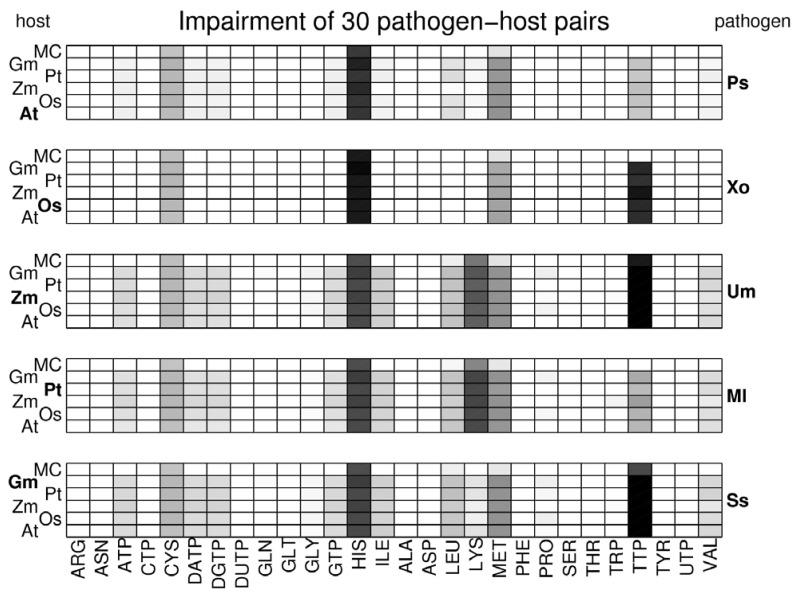
Relative impairment scores for essential biomass precursors for all investigated host-pathogen pairs. Impairment scores are indicated by grey-scale in a logarithmic scale. White squares indicate an impairment score of less than 0.01, black squares a score of 1. The network pairs are grouped by pathogens so that each panel displays the effect of one particular pathogen on each of the five plant networks and the complete MetaCyc network (MC). Native plant-pathogen pairs are highlighted in bold face.

To assess the negative impact of pathogens on the hosts, we calculated how the presence of the pathogens impairs the production of essential biomass precursors in the host networks (see Methods). To allow for a systematic analysis, we determined the impairment not only for the specific interactions of pathogens on their natural hosts, but extended the analysis to all combinations of host-pathogen pairs. Effectively, the non-natural plant-pathogen pairs produced *in silico* serve as a null-model to which any specific effects of the actual plant-pathogen combinations can be contrasted. The result is depicted in Figure 4 in which the shadings of the squares indicate the impairment scores in a logarithmic scale. A white square represents a score of less than 0.01, a black square of a score close to 1. 

Interestingly, only few biomass precursors show a high score while most precursors are only marginally impaired. Only for histidine, lysine, methionine and thymidine triphosphate (TTP) scores over 0.05 are observed. Histidine is strongly impaired in all investigated plant-pathogen pairs with scores between 0.25 and 0.85. Methionine and cysteine are also rather uniformly impaired in all pairs, but with considerably lower scores (between 0.05 and 0.08 for methionine and between 0.03 and 0.04 for cysteine). By contrast, the impairment of lysine and TTP is very heterogeneous throughout the investigated plant-pathogen pairs. While the production of thymidine triphosphate is strongly impaired by the pathogens *X. oryzae* (0.40-0.65), *U. maydis* (0.95-0.96) and *S. sclerotiorum* (0.99), it is only weakly impaired by the other two pathogens *P. syringae* (0.03) and *M. larici-populina* (0.03-0.06). This means that for *U. maydis* and *S. sclerotiorum* for almost all predicted nutrient combinations, removal of the nutrients leads to a disability of the host network to produce the essential nucleotide phosphate TTP. Interestingly, their metabolic networks exhibit a low pairwise distance (0.144) so it can be speculated that the mechanism is similar, despite the fact that *S. sclerotiorum* is a nectrotrophic parasite, while *U. maydis* is biotrophic. On the other hand, the network of *M. larici-populina*, the third fungal pathogen which is also biotrophic, also exhibits a low distance to *S. sclerotiorum* and *U. maydis*, but the impairment on host networks is considerably different. Similarly, lysine is considerably impaired by the pathogens *U. maydis* (0.18-0.21) and *M. larici-populina* (0.25-0.29), but only marginally by the other three pathogens (scores below 0.02). 

The observation that only a subset of biomass precursors is susceptible to impairment may result from a general, non-plant-specific, vulnerability of the respective synthesis pathways. To investigate the general fragility of these synthesis pathways, we have included the network comprising all reactions from MetaCyc [52] as a hypothetical host network. The impairment of the pathogens on the MetaCyc network (marked MC) is displayed in the top row of each panel in Figure 4. Histidine, for example, is as strongly impaired in the MetaCyc network as it is in the plant networks, suggesting that the full set of reactions does not provide a higher robustness for histidine synthesis when compared to the plant-specific synthesis pathways. Lysine production is impaired in the MetaCyc network only by the two pathogens *U. maydis* and *M. larici-populina*, which also impair lysine production in the plant networks. However, the considerably lower impairment scores (0.12 and 0.09, respectively) indicate that the full set of MetaCyc reactions provides more alternative synthesis routes and thus displays an increased robustness against competition by the pathogens. An interesting pattern is observed for the impairment of TTP. The pathogens *U. maydis* and *S. sclerotiorum*, which strongly impair TTP production in plant networks, also exhibit the strongest effect on the full network, albeit with a lower impairment score (0.64 and 0.27, respectively). By contrast, *X. oryzae*, which also strongly impairs TTP in plant networks, has only a negligible effect on the full network (<0.01). This indicates that in the case of *X. oryzae* other metabolic routes exist in MetaCyc, which could circumvent the removal of the required nutrients, while this is not the case for *U. maydis* and *S. sclerotiorum.*

**Figure 5:**
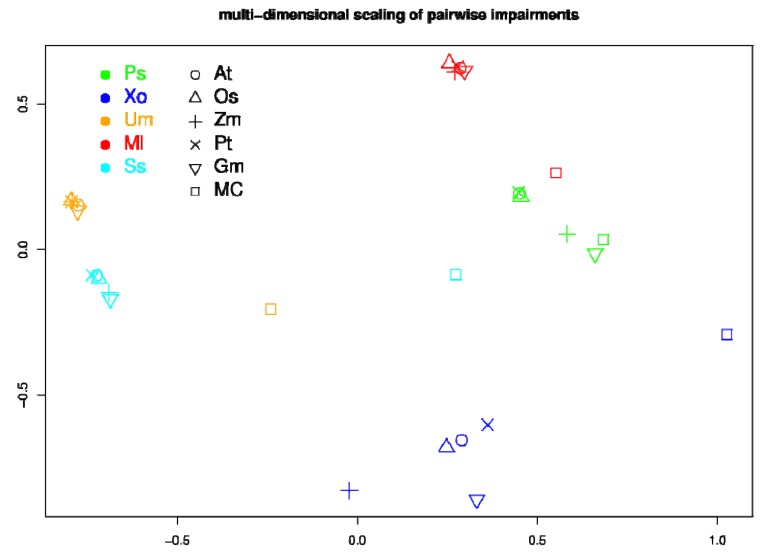
Multi-dimensional scaling (MDS) of all pairwise impairment patterns. Each colored symbol represents one host-pathogen pair, where hosts are characterized by different symbols and pathogens by different colors. Similar impairment patterns are located near each other in the plot. Axes denote the two-dimensional space in which the respective data points were placed by the MDS procedure.

As a general tendency, the impairment patterns appear to be largely determined by the pathogens and rather independent of the host species. For a systematic investigation of the similarities in the impairment patterns, we quantify the overall effect of a pathogen on a host by the vector containing as elements the 28 impairment scores for the biomass precursors and calculated the pairwise Manhattan (1-norm) distances. These distances were used to perform a multi-dimensional scaling [53] to visualize similarities and differences. The resulting plot (Figure 5) highlights that pairs containing the same pathogens (same colors) are always grouped together, confirming that the impairment pattern is largely determined by the pathogen. Furthermore, it can be observed that in most cases the impairment on the full MetaCyc network (squares) is clearly distinguished from the impairment on the plant networks. The only exception is *P. syringae*, which displays a similar effect on the full network as on the plant-specific networks. This exception can be explained by the fact that *P. syringae* mainly impairs the production of histidine. However, as discussed above, histidine production is almost equally impaired in the full MetaCyc network as in the plant-specific networks.

## 4. Discussion and Conclusions

The metabolic level constitutes an important layer of molecular organization as it is closely linked to the species’ phenotype. Here, we investigated the consequences of merging the metabolic networks of plants and their microbial pathogens mimicking pathogen attack of the respective plant. We explored the effects of an unrestricted exchange of the entire complement of all metabolites of two organisms as determined from genome-wide network reconstructions. As in reality the exchange of metabolites will be confined to a much smaller number of compounds due to the compound-specific transport across biomembranes, the approach presented here can be seen as a limiting case of plant-pathogen interactions. Our study constitutes a first attempt to gauge the negative and positive effects of joint metabolic networks between plants and their microbial phytopathogens.

Homology based metabolic network reconstruction has been applied successfully to many organisms [54,55]. For a newly sequenced genome, this method is the most direct strategy to explore the biochemical repertoire of an organism. One limitation for this method is that for a substantial number of enzymes, no sequence information exists. For 43% of all MetaCyc reactions, no DNA/protein sequences are presently available. As a consequence, important reactions may be missed. Modern high-throughput metabolic profiling experiments can possibly mitigate the limitations from this missing information, because it is a plausible assumption that for every observed metabolite enzymes utilizing it as a substrate or producing it must exist [56]. Thus, the presence of a particular enzymatic activity can be postulated even in the absence of sequence information. Inevitably, a sequence homology based transfer of functional annotation relies on sequence identity-cutoffs, which may either be overly restrictive - leading to unnecessarily small networks - or too permissive - leading to many false positive enzyme annotation. Confirmation of the annotated functions can only be achieved by experimental enzyme assays. 

Inevitably, these uncertainties also apply to the networks reconstructed for this study. In principle, for some of the organisms investigated here, well-curated metabolic networks exist, such as AraCyc [57] for *A. thaliana*. However, we decided against using the curated networks, but applied the same annotation workflow used for the other organisms for which no pre-existing metabolic database was available. Thereby, possible methodological and systematic differences when comparing results for the different species were avoided. Our method was based on deriving HMMs for every enzyme-catalysed reaction and to apply those models in whole-genome scans. In the case of *A. thaliana*, this resulted in a high number of consistent annotations compared to AraCyc, but also yielded annotations for which no corresponding enzyme annotation was available. Specifically, of the 3316 reactions present in our Arabidopsis network, 1764 were also present in AraCyc, 1552 were contained in our set only, and 1556 were unique to AraCyc. The latter set included almost exclusively those reactions for which no corresponding enzyme information was available. Thus, they cannot be contained in our set. Reactions contained in our set only were all statistically significant and represent either alternative annotations to enzymes for which another EC number with different substrate specificity was already assigned, or they were not yet annotated previously in AraCyc. The comparison with AraCyc highlights the importance to apply the same annotation pipeline to all organisms to ensure consistency. It further illustrates that different annotation pipelines (AraCyc has not been constructed using HMMs) result in quite different networks, pointing at possible future approaches to improve current Biocyc databases.

Our network reconstruction workflow included a gap filling step (Table 3), in which enzymatic reactions were added that are essential in the sense that all biomass precursors must be producible from the available nutrients. This was only possible for the plant networks as for the pathogens, defined culture media are not known. It is, however, conceivable that the missing enzymes are contributed by a symbiont as, for example, reported for carotenoid synthesis in the whitefly *Bemisia tabaci* by its endosymbiont *Portiera aleyrodidarum* [58]. In fact, two of the four added reactions in *Glycine max* exist in its pathogen *Sclerotinia sclerotiorum*, one involved in the histidine biosynthesis and the other in inositol-5-phosphate. In the four added reactions in *Zea mays*, one reaction exists in its pathogen *Ustilago maydis,* also involved in inositol-5-phosphate biosynthesis. However, without the added reactions, the plant networks would not have been able to photoautotrophically accumulate biomass, which contradicts our biological knowledge as the plants studied here can live without the pathogen. Unless the plants live in an obligate symbiosis, reactions that need to be present in the plants for their self-sustained growth have to be added to the plant network. For the added reactions of the other three plants, there is no equivalent reaction existing in their pathogens.

There are several powerful tools and resources for visualization of genome-wide metabolic networks based on a manually drawn network map [48,59]. However, there is presently no tool specifically designed to visualize host-pathogen metabolic networks. Here, we exploited the KEGG mapping functionalities to accomplish this task. We thus provide the means to biochemists and microbiologists to easily and visually inspect the interaction of two metabolic networks of a plant host and a disease-causing parasite.

The targeted reconstruction of pairs of networks representing the metabolism of a pathogen and its host is a prerequisite for future theoretical analyses of their interaction at the metabolic level. While visual inspection is highly useful, especially for the expert with the trained eye, many features and properties are far from obvious and can only be uncovered by computational analyses. Here, we presented two approaches to how the interaction of metabolic networks can be quantified. A positive, synergistic effect is captured by the metabolic gain previously defined in [20]. To this end, we calculated how many new metabolites might be produced from carbon dioxide and inorganic nutrients in a combined network consisting of all reactions of the host and the pathogen. We found that this number varies considerably (from 1 in the pair *At*/*Ps* to 191 for *Gm/Ss*) and does not appear to be correlated with the dissimilarity of the network pairs as judged by the Jaccard distance. To estimate how the availability of producing necessary biomass precursors and, therefore, growth is impaired by the presence of another organism, we introduced here as a new measure the 'metabolic impairment'. Our analysis is unbiased in the sense that it only uses the reconstructed metabolic networks, but not any other prior biological information except that plants alone can grow on carbon dioxide as the only carbon source. From the pathogen network, many different possible nutrient combinations, which can sustain growth, are computed and the effect of removing these nutrients from the host network on the host's capability to grow is determined. We considered the nutrient drained by the pathogen completely absent to the plant. Obviously, this may not be the case in reality. However, this assumption allowed for the simplest treatment of the problem and can furthermore be seen as an extreme and limiting case. When investigating the impairment in all 25 plant-pathogen pairs (Supplementary Material 3), we found that the amino acids histidine, lysine, and to a lesser extent methionine, as well as the nucleotide phosphate TTP are particularly vulnerable in all investigated pairs; *i.e.* the pathogens were found to frequently remove compounds that are essential for their production in plants. Whether this finding can be explained by intrinsic properties of metabolism in general is not yet clear. However, a comparison with the negative effect on the complete MetaCyc network indicated that intrinsic network properties are at least partially responsible for the dominating appearance of certain biomass precursors. Interestingly, also in a different unbiased network analysis TTP stood out among the nucleotide phosphates. In [60], it was found that the metabolic scope of TTP in the global network comprising all reactions known to date was considerably smaller than that of the other deoxy-nucleotides, which essentially means that, while TTP can be produced from any other nucleotide and water as sole substrates, the reverse is not possible. A robustness analysis of the *E. coli* network [47] has also illustrated that TTP, methionine, and histidine are particularly vulnerable if reactions are randomly deleted from the network. 

A key finding of this study was that the impairment pattern is largely determined by the pathogen. For every pathogen, the impairment scores for the different biomass precursors were similar regardless with which of the five plants the pathogen was paired. As three of the five selected pathogens were fungi and two bacteria, the results can also be analyzed with regard to kingdom-specific effects. However, no strong segregation of fungi and bacteria was evident (Figure 5). Clearly, the number of representatives (3 and 2 species, respectively) is too low to allow definitive statistical conclusions. With an increased availability of more fully sequenced plant species and associated pathogen genomes, the question of kingdom-specific effects needs to be revisited. Similarly, for conclusions with regard to life-style characteristics of the pathogens, more network pairs will be necessary. Nonetheless, we believe that the current study introduces appropriate theoretical concepts for the investigation of plant-pathogen metabolic network effects. An interesting extension of our investigation would be to pair a large number of networks including pathogens and non-pathogens with the host networks and repeat the impairment analysis. If the producibility of the same metabolites is particularly impaired also in such a randomized approach, the hypothesis that some intrinsic features of the amino-acid synthesizing pathways are responsible for our observation is supported.

The approaches presented here may provide valuable insight into possible mechanisms of how pathogens exploit their hosts and on which particular metabolites they depend. Such hypotheses generated by our modeling approach are in principle testable by experimental techniques. For example, metabolite exchange fluxes between host and parasite can in principle be measured using isotope labeling techniques. As more such information becomes available, the network analysis must be further refined. To this end, it will become necessary to generate highly curated metabolic network models for the purpose of performing constraint-based modeling such as Flux Balance Analysis (FBA, [61]). With these models, flux distributions can be predicted, which for example lead to a maximal biomass production of the pathogen and experimental validation or falsification will lead to a continuous improvement of the metabolic models and our understanding on the metabolic exchanges between plants and phytopathogens.

## Supplementary Material

(1) Navigable metabolic network maps for the plant-pathogen pairs At-Ps, Gm-Ss, Os-Xo, Pt-Ml, and Zm-Um.
(2) The reactions list for all the species used in this research and MetaCyc.
(3) Various plant-pathogen impairment profile plots.

Supplementary File 1 Supplementary Material 1 (ZIP, 7297 KB)

Supplementary File 2 Supplementary Material 2 (ZIP, 146 KB)

Supplementary File 3 Supplementary Material 3 (ZIP, 684 KB)
