# Supplementary figures and images for: The Metabolic Interplay between Plants and Phytopathogens
Source: Metabolites. 2013 Jan 8;3(1):1–23. doi: 10.3390/metabo3010001 (PMC3901261; doi:10.3390/metabo3010001)

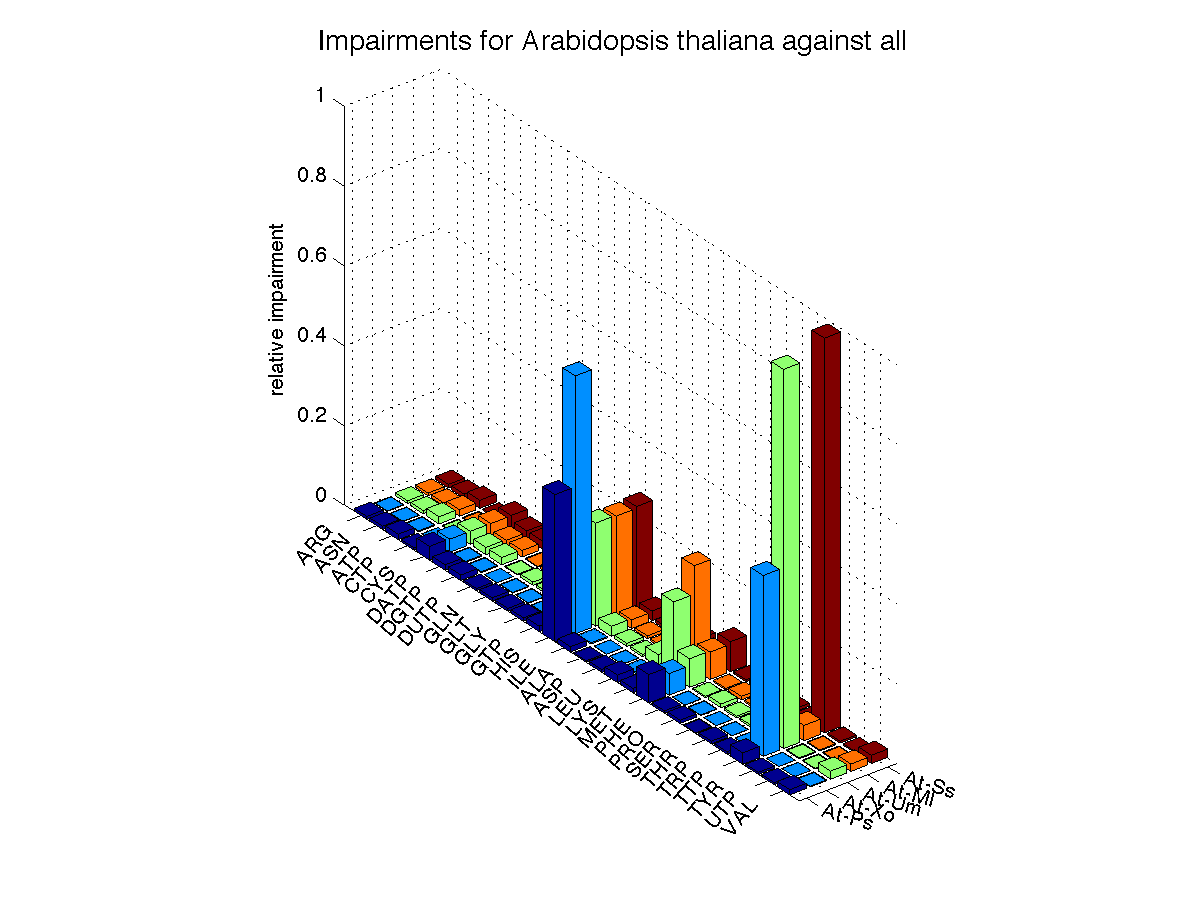

Supplement: Supplementary File 3 — Supplementary Material 3 (ZIP, 684 KB) [file metabolites-03-00001-s003.zip › impairmentFigures/impairmentAt.png]

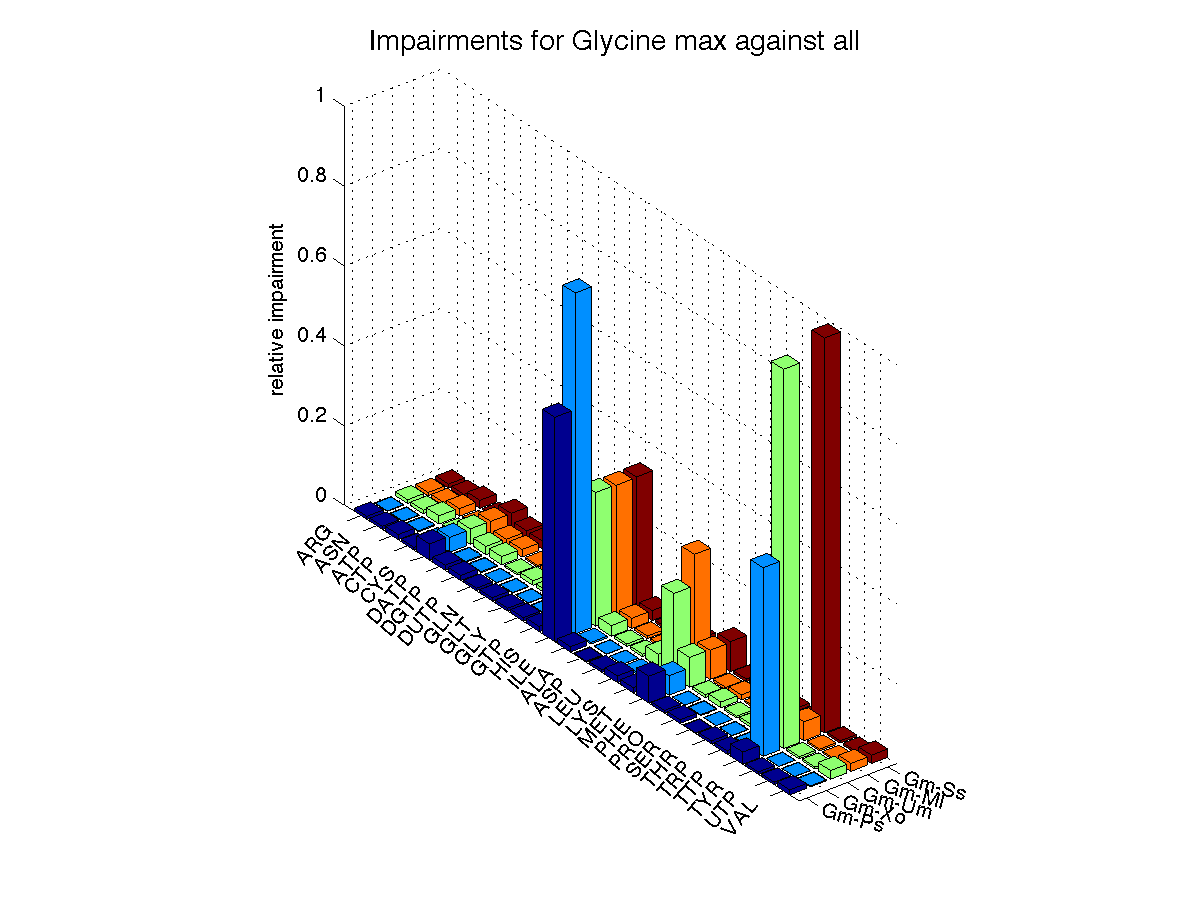

Supplement: Supplementary File 3 — Supplementary Material 3 (ZIP, 684 KB) [file metabolites-03-00001-s003.zip › impairmentFigures/impairmentGm.png]

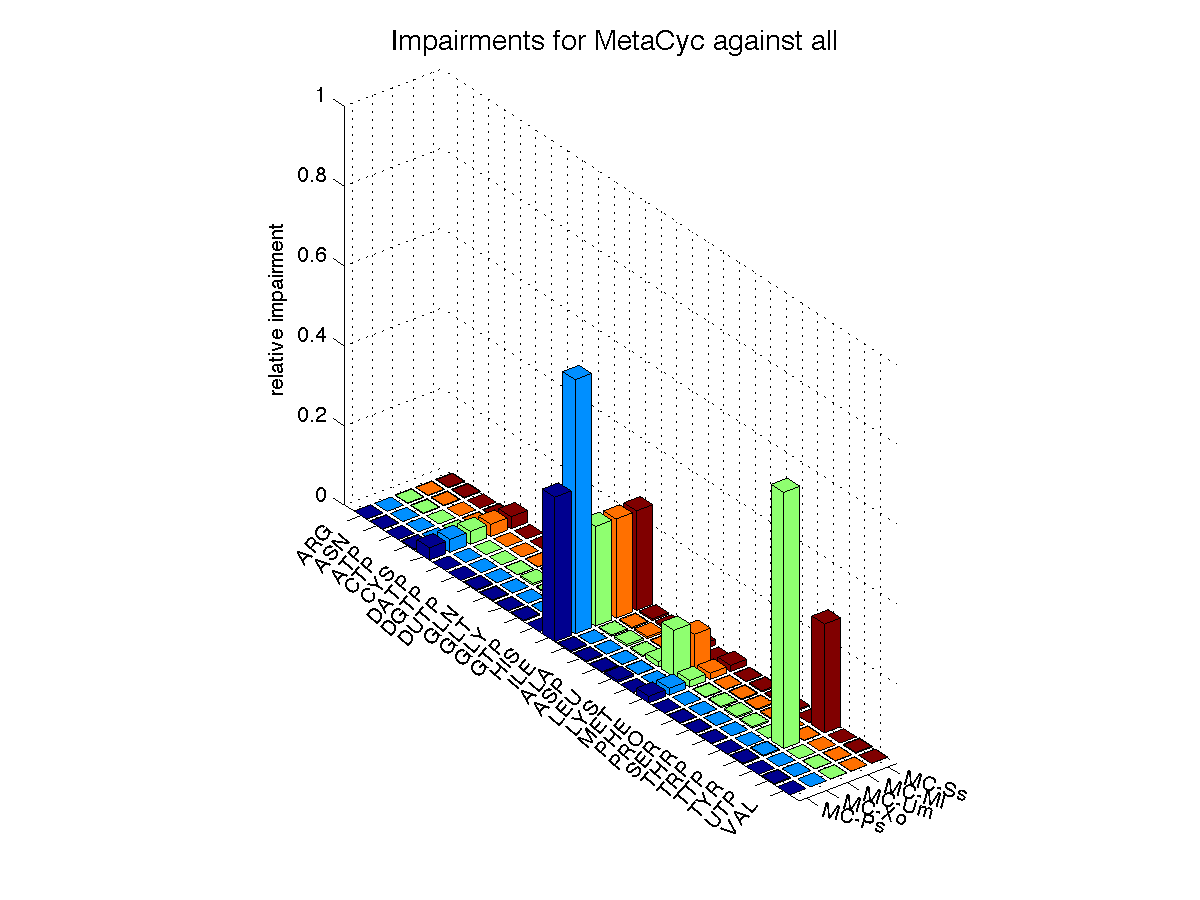

Supplement: Supplementary File 3 — Supplementary Material 3 (ZIP, 684 KB) [file metabolites-03-00001-s003.zip › impairmentFigures/impairmentMC.png]

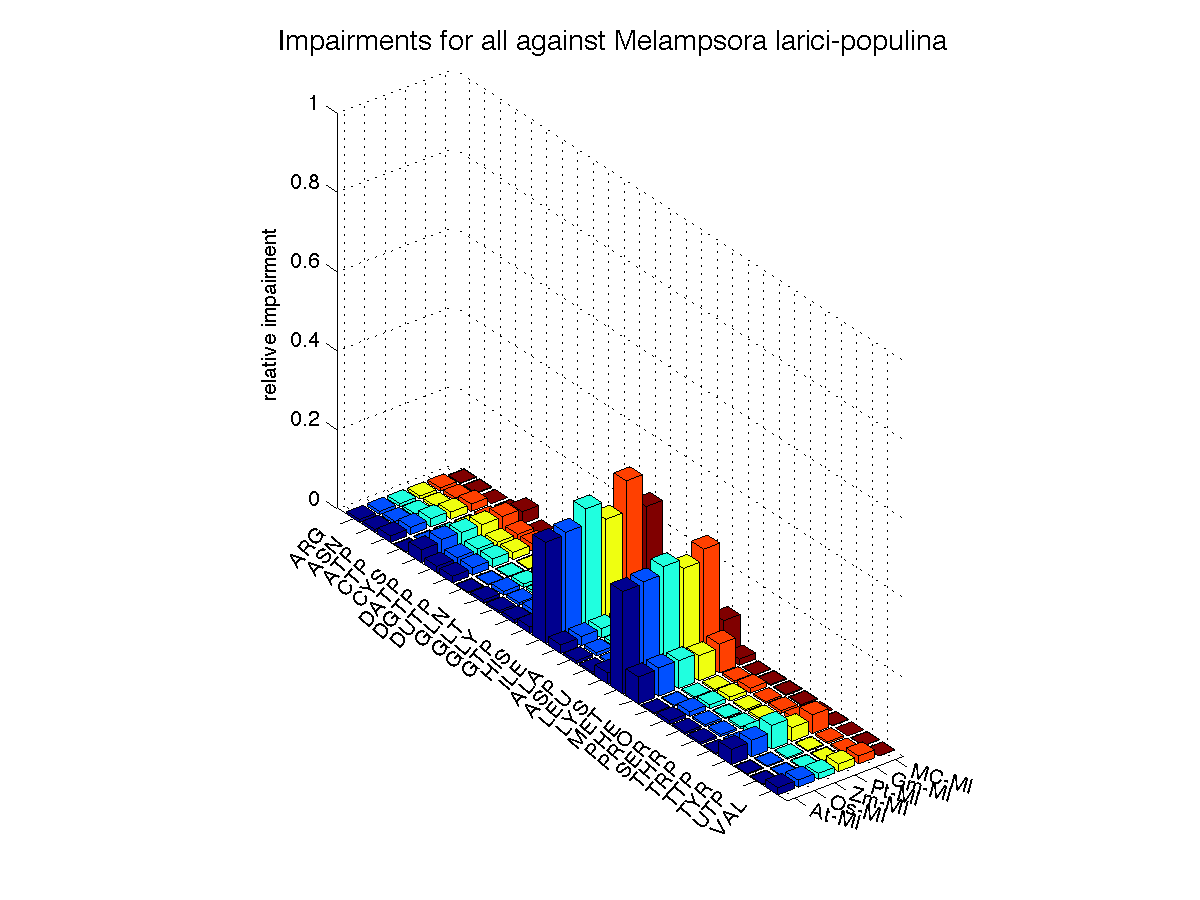

Supplement: Supplementary File 3 — Supplementary Material 3 (ZIP, 684 KB) [file metabolites-03-00001-s003.zip › impairmentFigures/impairmentMl.png]

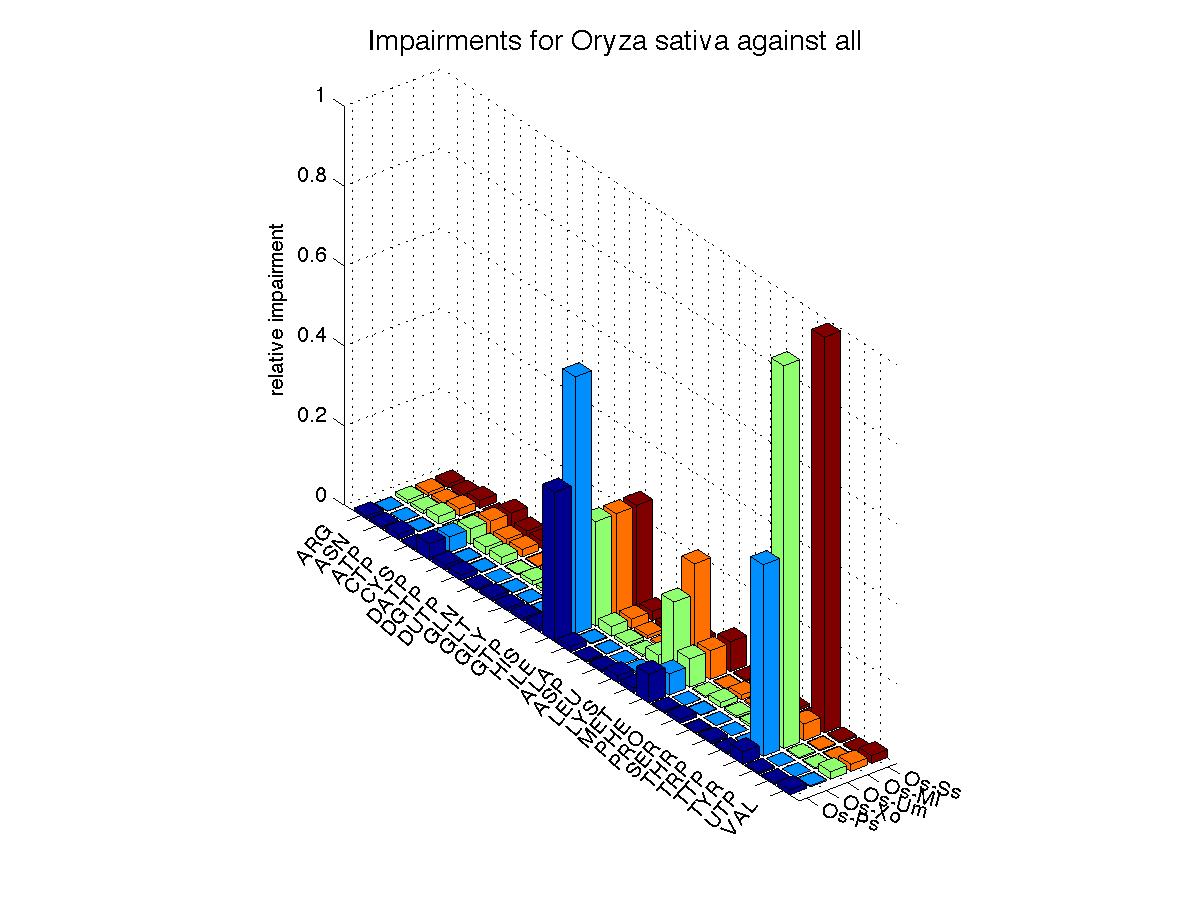

Supplement: Supplementary File 3 — Supplementary Material 3 (ZIP, 684 KB) [file metabolites-03-00001-s003.zip › impairmentFigures/impairmentOs.png]

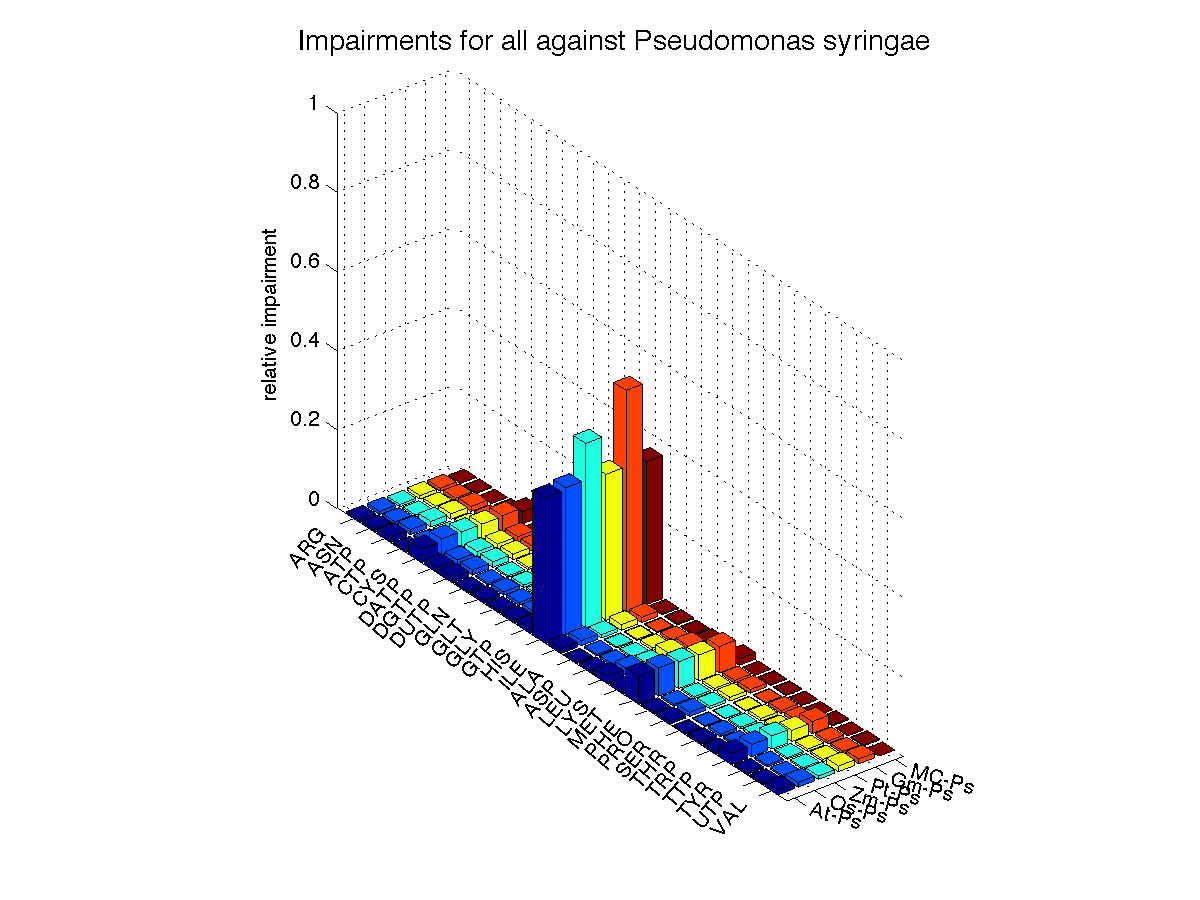

Supplement: Supplementary File 3 — Supplementary Material 3 (ZIP, 684 KB) [file metabolites-03-00001-s003.zip › impairmentFigures/impairmentPs.png]

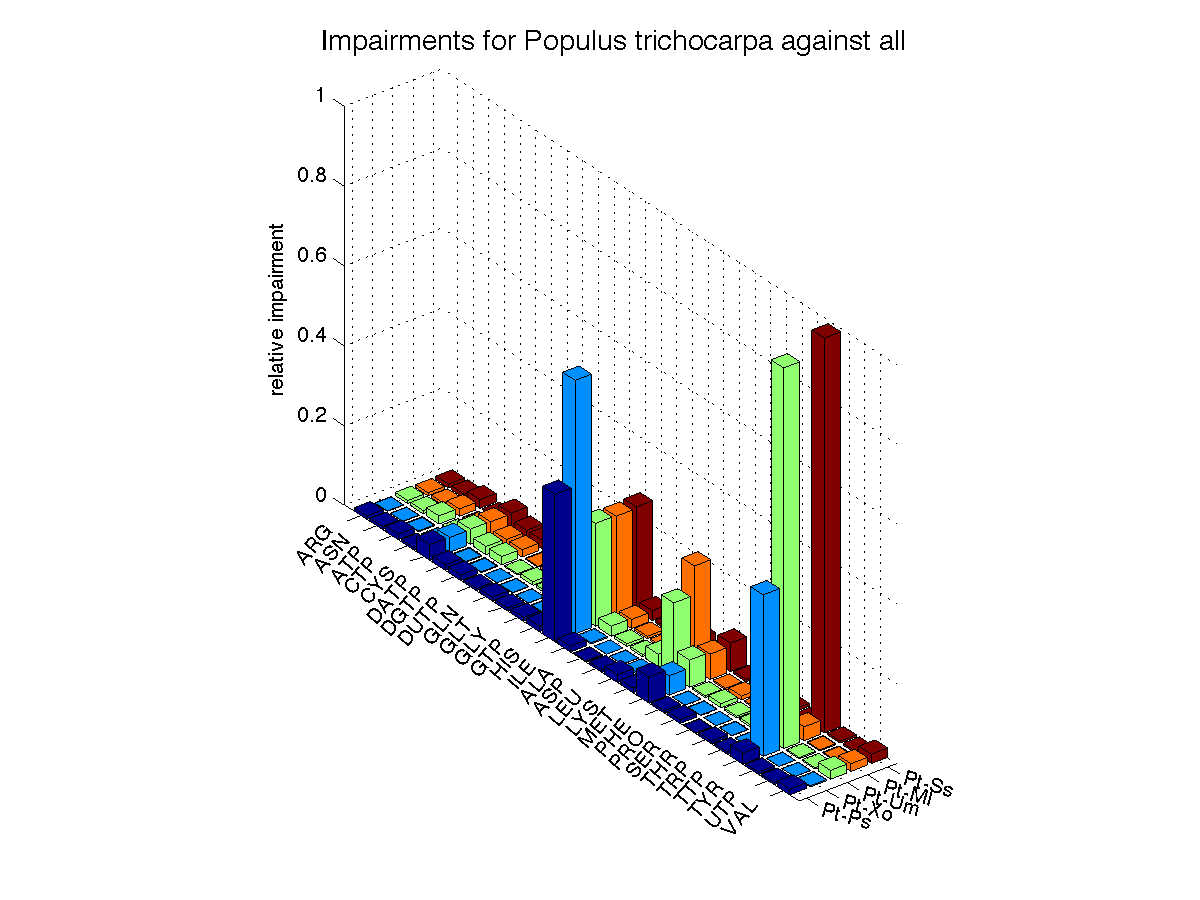

Supplement: Supplementary File 3 — Supplementary Material 3 (ZIP, 684 KB) [file metabolites-03-00001-s003.zip › impairmentFigures/impairmentPt.png]

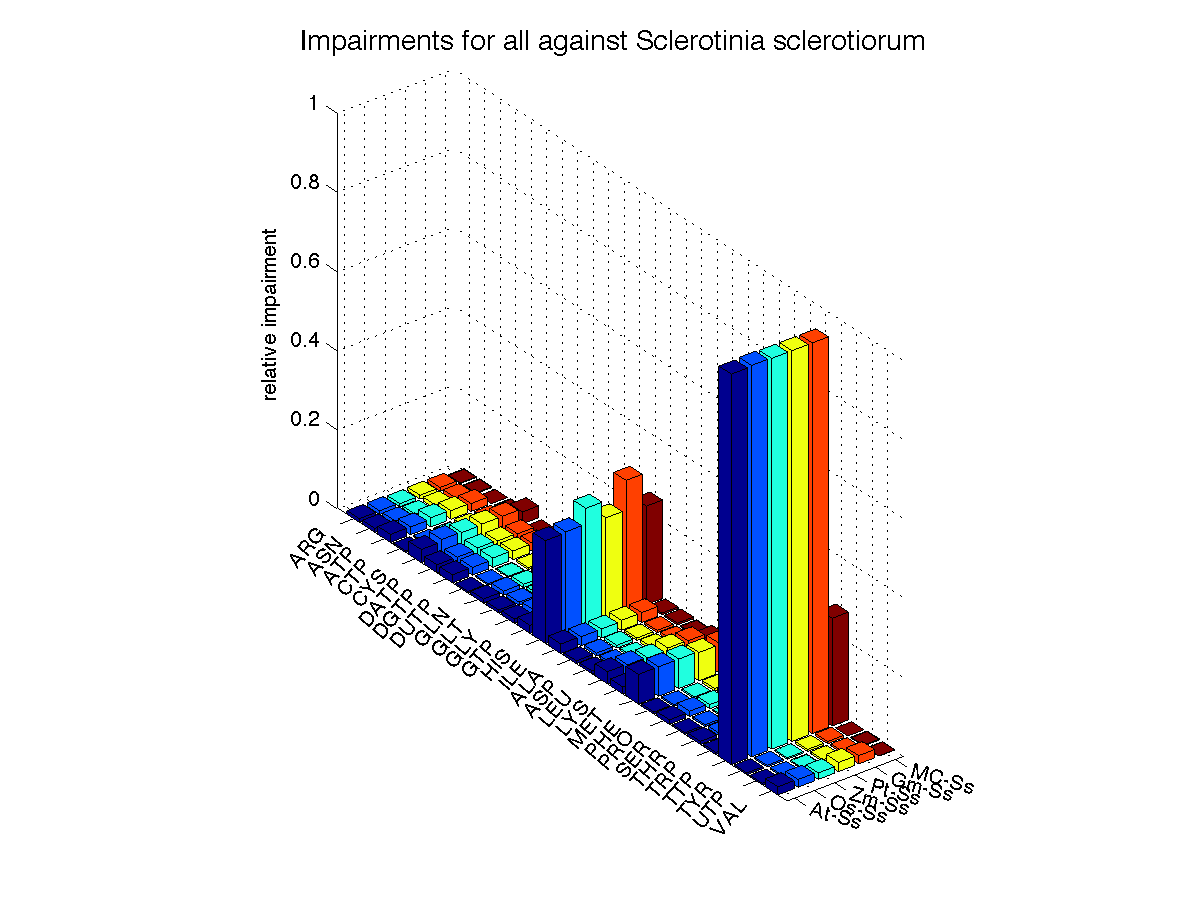

Supplement: Supplementary File 3 — Supplementary Material 3 (ZIP, 684 KB) [file metabolites-03-00001-s003.zip › impairmentFigures/impairmentSs.png]

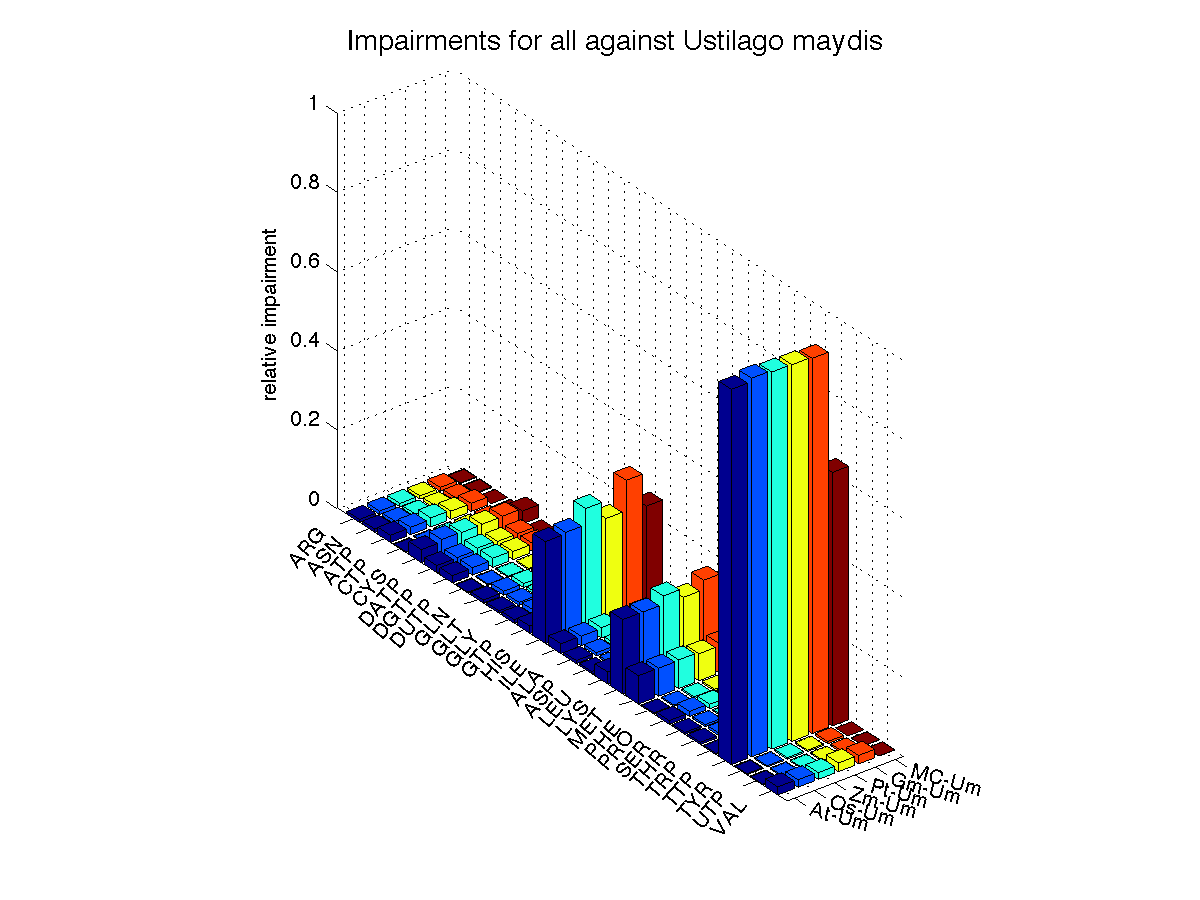

Supplement: Supplementary File 3 — Supplementary Material 3 (ZIP, 684 KB) [file metabolites-03-00001-s003.zip › impairmentFigures/impairmentUm.png]

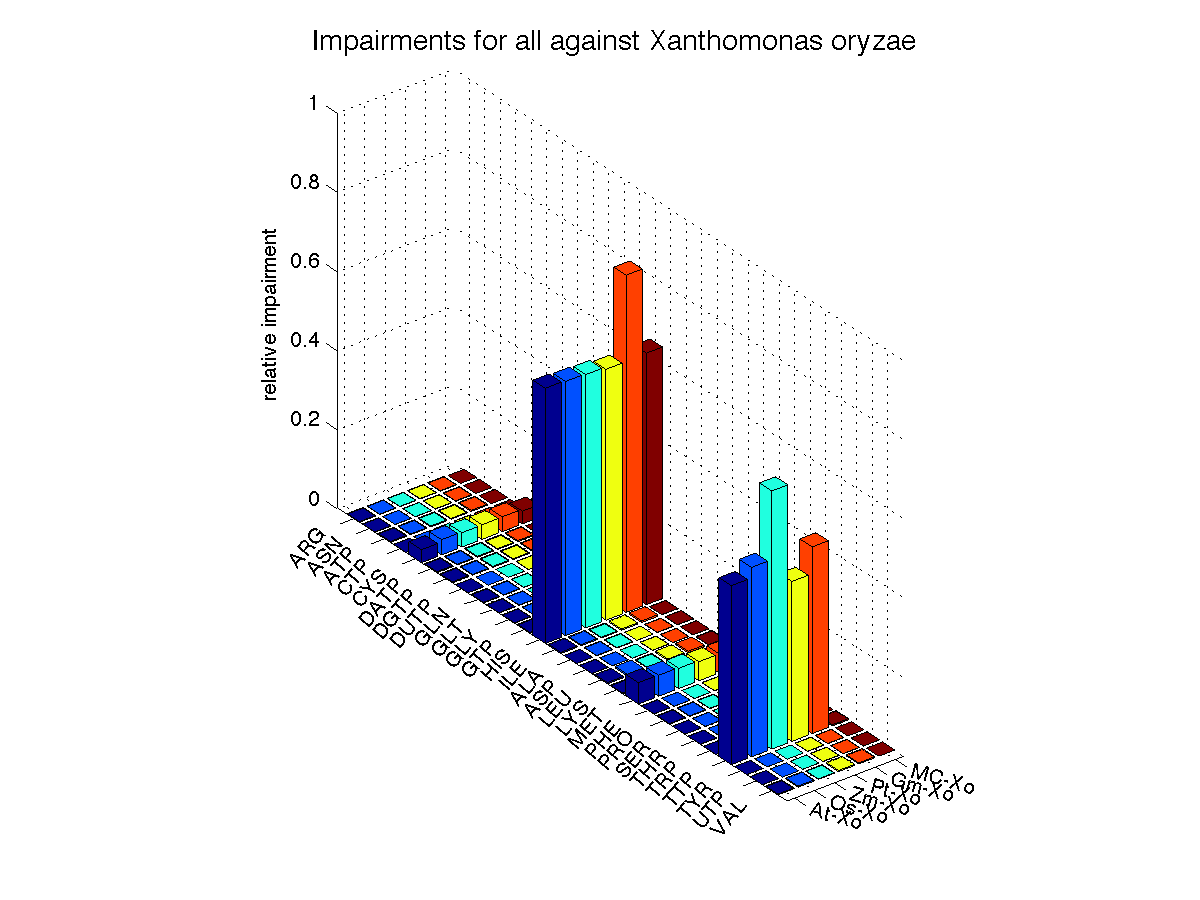

Supplement: Supplementary File 3 — Supplementary Material 3 (ZIP, 684 KB) [file metabolites-03-00001-s003.zip › impairmentFigures/impairmentXo.png]

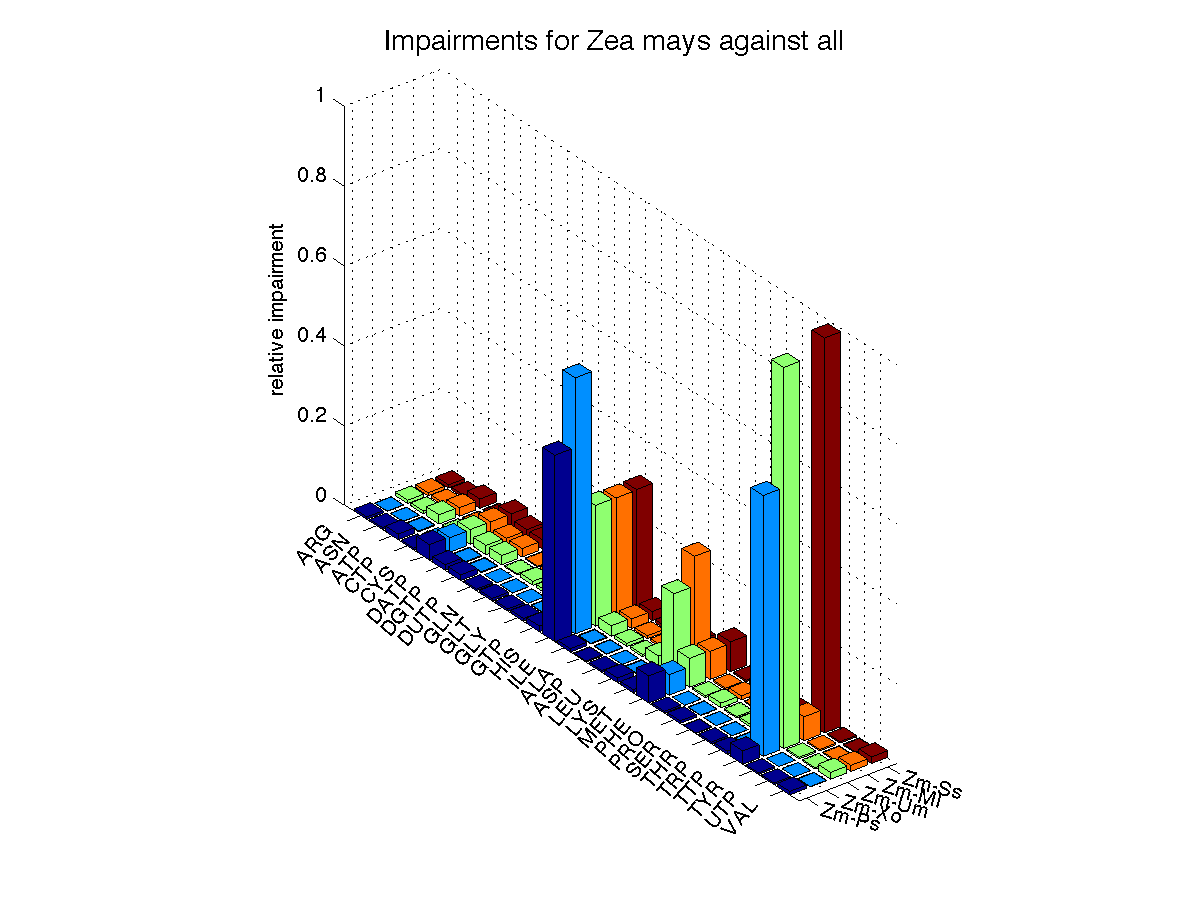

Supplement: Supplementary File 3 — Supplementary Material 3 (ZIP, 684 KB) [file metabolites-03-00001-s003.zip › impairmentFigures/impairmentZm.png]
